# The Application of Non-Invasive Apoptosis Detection Sensor (NIADS) on Histone Deacetylation Inhibitor (HDACi)-Induced Breast Cancer Cell Death

**DOI:** 10.3390/ijms19020452

**Published:** 2018-02-02

**Authors:** Kai-Wen Hsu, Chien-Yu Huang, Ka-Wai Tam, Chun-Yu Lin, Li-Chi Huang, Ching-Ling Lin, Wen-Shyang Hsieh, Wei-Ming Chi, Yu-Jia Chang, Po-Li Wei, Shou-Tung Chen, Chia-Hwa Lee

**Affiliations:** 1 Research Center for Tumor Medical Science, China Medical University, Taichung 40402, Taiwan; kwhsu@mail.cmu.edu.tw; 2 Graduate Institutes of New Drug Development, China Medical University, Taichung 40402, Taiwan; 3 Department of Surgery, School of Medicine, College of Medicine, Taipei Medical University, Taipei 11031, Taiwan; cyh@tmu.edu.tw (C.-Y.H.); kelvintam@tmu.edu.tw (K.-W.T.); r5424012@tmu.edu.tw (Y.-J.C.); poliwei@tmu.edu.tw (P.-L.W.); 4 Division of General Surgery, Department of Surgery, Shuang Ho Hospital, Taipei Medical University, New Taipei City 23561,Taiwan; 5 Institute of Bioinformatics and Systems Biology, National Chiao Tung University, Hsinchu 30068, Taiwan; chunyulin.bi99g@g2.nctu.edu.tw; 6 Bioinformatics Center, Institute for Chemical Research, Kyoto University, Kyoto 611-0011, Japan.; 7 Department of Endocrinology and metabolism, Cathay General Hospital, Taipei 10630, Taiwan; likih@seed.net.tw (L.-C.H.); cheering@tmu.edu.tw (C.-L.L.); 8 Department of Laboratory Medicine, Shuang Ho Hospital, Taipei Medical University, Taipei 23561, Taiwan; 12638@s.tmu.edu.tw (W.-S.H.); rc4202@tmu.edu.tw (W.-M.C.); 9 Graduate Institute of Clinical Medicine, College of Medicine, Taipei Medical University, Taipei 11031, Taiwan; 10 Division of Colorectal Surgery, Department of Surgery, Wan Fang Hospital, Taipei Medical University, Taipei 11696, Taiwan; 11 Division of Colorectal Surgery, Department of Surgery, Taipei Medical University Hospital, Taipei Medical University, Taipei 11031, Taiwan; 12 Cancer Research Center and Translational Laboratory, Department of Medical Research, Taipei Medical University Hospital, Taipei Medical University, Taipei 11031, Taiwan; 13 Graduate Institute of Cancer Biology and Drug Discovery, Taipei Medical University, Taipei 11031, Taiwan; 14 Comprehensive Breast Cancer Center, Changhua Christian Hospital, Changhua 50006, Taiwan; 15 School of Medical Laboratory Science and Biotechnology, College of Medical Science and Technology, Taipei Medical University, Taipei 11031, Taiwan; 16 Ph.D. Program in Medicine Biotechnology, College of Medicine, Taipei Medical University, Taipei 11031, Taiwan; 17 Comprehensive Cancer Center of Taipei Medical University, Taipei 11031, Taiwan

**Keywords:** live cell non-invasive apoptosis detection sensor (NIADS), triple negative breast cancer (TNBC), HDACi

## Abstract

Breast cancer is the most common malignancy in women and the second leading cause of cancer death in women. Triple negative breast cancer (TNBC) subtype is a breast cancer subset without ER (estrogen receptor), PR (progesterone receptor) and HER2 (human epidermal growth factor receptor 2) expression, limiting treatment options and presenting a poorer survival rate. Thus, we investigated whether histone deacetylation inhibitor (HDACi) could be used as potential anti-cancer therapy on breast cancer cells. In this study, we found TNBC and HER2-enriched breast cancers are extremely sensitive to Panobinostat, Belinostat of HDACi via experiments of cell viability assay, apoptotic marker identification and flow cytometry measurement. On the other hand, we developed a bioluminescence-based live cell non-invasive apoptosis detection sensor (NIADS) detection system to evaluate the quantitative and kinetic analyses of apoptotic cell death by HDAC treatment on breast cancer cells. In addition, the use of HDACi may also contribute a synergic anti-cancer effect with co-treatment of chemotherapeutic agent such as doxorubicin on TNBC cells (MDA-MB-231), but not in breast normal epithelia cells (MCF-10A), providing therapeutic benefits against breast tumor in the clinic.

## 1. Introduction

Epigenetic modifications play a crucial role in chromatin remodeling of cancer hallmarks [1]. The term “epigenetic” refers to gene expression change without DNA sequence substitution. DNA methylation and posttranslational histone modifications are the two main epigenetic modifications in humans [2], with histone modifications consisting of acetylation, methylation, and phosphorylation processes. Histone acetylation has also been considered as one of the most important epigenetic regulations in gene expression, whereas acetylation and deacetylation of histone act as a switch for gene activation and repression, respectively. Eukaryotic chromatin is composed of repeating nucleosomes, which are wrapped around a core octamer of eight histone protein subunits. Every individual nucleosome has two pairs of different histone proteins, H2A, H2B, H3 and H4, connected as H2A-H2B dimers and an (H3)_2_(H4)_2_ tetramer, respectively. When histone is acetylated by histone acetyl-transferases (HATs) on the tail end, histones become negatively charged and are associated with a looser chromatin state and gene-transcription activation. On the other hand, histone acetylation is modulated by histone deacetylases (HDACs) and is associated with a tighter chromatin state and gene transcriptional silencing [3]. This type of epigenetic regulation plays a pivotal role in tumor biology, as tumor cells tend to manipulate selective gene expression in order to facilitate survival [1,4].

Overexpression of HDAC proteins is frequently found in numerous cancers [5]. Thus, using HDAC inhibitors (HDACi) to increase acetylation of cellular proteins by abrogating HDAC activity and reversing the malignancy has emerged as a promising anticancer therapy [1,4]. According to the previous study, even though a similar level of acetylated histones has been shown in both normal and tumor cells with HDACi exposure, normal cells appear relatively resistant to the HDACi treatment, thereby reducing any side effects that might have occurred [6]. All these characteristics seem to confirm the strong potential of using HDACi as a therapeutic cancer treatment in future clinics.

Breast cancer is the most common malignancy in women and the second leading cause of cancer death in women, after lung cancer [7]. For breast cancer therapy, breast cancer is divided into several subtypes based on immunohistochemical markers such as the estrogen receptor (ER), progesterone receptor (PR), and HER2/*neu* expressions [8]. Basal-like or triple negative breast cancer (TNBC) subtype is a histological breast cancer subset without expression of these receptors, limiting treatment options and presenting a poorer survival rate. TNBC represents only 15–20% of patients with breast cancer. The poor prognosis of TNBC may be due to its unique histological features, such as its high grade, high proliferative rate, and low apoptotic cells [9]. All these pathological features make TNBC still the most aggressive tumor subtype with limited clinical therapy. More recently, three clinical trials reported in the American Society of Clinical Oncology (ASCO) meeting of 2016 using new targeted therapies have presented successful results against triple negative breast cancer. These studies target Trop2 [9], frizzled receptor and PD-L1 [10,11] oncoproteins in combination with chemotherapy paclitaxel, exhibiting great potential to extend the lives of TNBC patients whose cancers have progressed after previous treatments. However, intense research is still ongoing to identify specific biomarkers and develop additional and effective treatment options. Until then, different investigation aspects of TNBC biology will help us to evaluate novel, specific approaches dedicated to this hard-to-treat disease.

In this study, we investigated whether HDACi could be used as a potential anti-cancer therapy on breast cancer cells. More importantly, the specific subtype of breast cancers which are sensitive to four FDA-approved HDACi will be identified in detail, and cytotoxicity on normal breast epithelial cells will also be measured. On the other hand, we developed a bioluminescence-based live cell apoptosis detection assay by split-luciferase fragment system through lentivirus transfection. The powerful combination of lentivirus transfection and non-invasive apoptosis detection sensor (NIADS) detection has the advantage of being easy to handle and performing the quantitative and kinetic analyses of apoptotic cell death by HDAC or anti-cancer drugs on cells, compared to other apoptosis detection assays such as apoptotic protein activation, flow cytometry and LIVE/DEAD cell assays. In addition, the use of HDACi may also be accompanied with another effect that enhances drug sensitivity during chemotherapeutic protocols, providing therapeutic benefits against breast tumor in the clinic.

## 2. Result

### 2.1. Development of Lentivirus Mediates Non-Invasive Caspase-3 Reporter Assay

**Figure 1:**
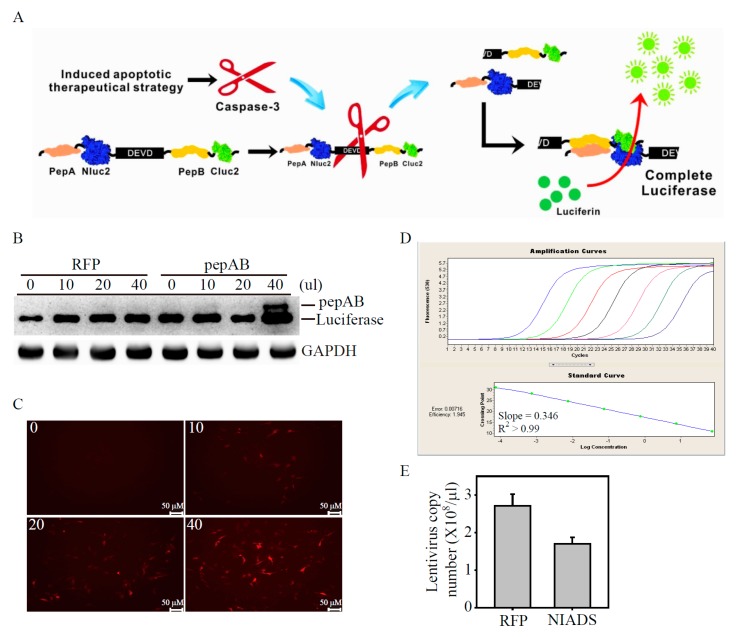

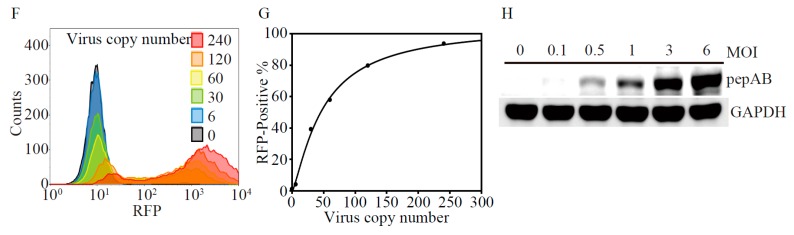
Optimization of virial transduction conditions for human MDA-MB-231 cells (**A**) Schematic representation of the non-invasive apoptosis detection sensor (NIADS). The fusion proteins of pepA-Nluc and pepB-Cluc fragment were linked with 3Xcaspase-3 cleavage sequences (DEVD). Once the cell is undergoing the apoptosis event, activated caspase-3 recognizes the DEVD sequence and cuts the fusion protein into two fragments. The proteins of pepA and pepB are known to have strong association force and therefore enable reconstitution of full-length luciferase. The bioluminescence activity can be further detected with substrate (Luciferin) addition; (**B**) NIADS DNA sequence was cloned into lentivirus plasmid and generated virus particles in medium. The virus titration from 10–40 μL of both RFP (red fluorescent protein) or NIADS was added in the culture medium of luciferase stable expressing MDA-MB-231 cells for three days. The cells were harvested and immunoblotted to luciferase antibody, whereas the full length of firefly luciferase was determined as protein loading control; (**C**) The virus titration from 10 to 40 μL RFP virus-containing medium was measured on MDA-MB-231 cells for three days. The image of RFP expression was captured under florescence microscopy. Scale bar: 50 μM; (**D**) Absolute qPCR analysis was measured by serial copy number of GUS (β-glucuronidase)-containing TA vector from a range of 10^2^ (purple line) to 108 (blue line)/μL as a standard curve. The gene amplification curves were also determined for standard error and gene amplification efficiency. The qPCR efficiency analysis for standard curve is calculated by the formula of Efficiency = 10^−1/slope^, whereas qPCR unexplained variance of R^2^ value was calculated by the formula of R^2^ = 1 − Error; (**E**) The purified and concentrated RFP and NIADS lentivirus were determined as virus copy number/μL by absolute qPCR analysis. Data is presented as the mean and standard error; (**F**) The purified and concentrated RFP lentivirus were measured as virus copy number by qPCR analysis. MDA-MB-231 cells were seeded in a 6-cm dish and infected with 6-, 12-, 30-, 60-, 120-, 240-fold concentrations of virus to MDA-MB-231 cell number for three days. The RFP-positive (infected) cell population was assessed using flow cytometry; (**G**) Linear curve comparison of virus input and the RFP-positive cell population; (**H**) The MOI (240-fold as MOI = 1, multiplicity of infection) from 0.1 to 6 of NIADS contain lentivirus were used on MDA-MB-231 cell. The NIADS fusion protein was immunoblotted by luciferase antibody.

Successful drug treatment in human cancers requires the therapeutic goal of triggering tumor-selective cell death, whereas apoptosis offers advantages over non-apoptotic death mechanisms only if the therapeutic index or the availability of compounds that induce it is greater [12]. However, it is a time-consuming and requires a great deal of labor to perform apoptosis analysis on anti-cancer drug screening. In order to develop a rapid and reliable biosensor for apoptosis detection, we constructed a fusion protein of luciferase fragments (Nluc and Cluc) that contains peptide A (pepA) and peptide B (pepB) at the amino termini with 3X repeats of caspase-3 cleavage sequences (DEVD), named the non-invasive apoptosis detection sensor (NIADS, Figure 1A). Upon induction of apoptosis and caspase-3 activation, cleavage at the DEVD site would free both pepA-Nluc and pepB-Cluc fragments and enable reconstitution of full-length luciferase by strong association of pepA and pepB peptides, resulting in bioluminescence activity from NIADS with substrate addition. The core sequence of this NIADS was transferred into lentivirus for better transfection efficiency and more flexible usage for apoptosis detection. In other words, the NIADS theoretically allows us to monitor caspase-3 status by measuring bioluminescence activity on cells or tumors. To ensure the lentivirus mediates NIADS and would transfect cells and produce NIADS, we infected different concentrations of RFP and NIADS lentivirus on luciferase expressed MDA-MB-231 cells. Here, RFP lentivirus was used as negative control, whereas native luciferase in MDA-MB-231 cells was used for comparing the molecular weight of NIADS fusion protein. In Figure 1B, NIADS fusion protein was strongly expressed in the treatment of 40 μL NIADS lentivirus-containing medium, whereas the RFP lentivirus-containing medium had no NIADS fusion protein expression, using luciferase against antibody. On the other hand, MDA-MB-231 cells express stronger RFP fluorescent proteins by the transfection of increasing RFP lentivirus-containing medium, illustrating the success of virus infection (Figure 1C). In order to standardize the lentivirus infection in each experiment, we used absolute qPCR analysis to measure the virus copy members of the concentrated RFP and NIADS lentivirus, according to the reference gene analysis (Figure 1D). The absolute qPCR efficiency and unexplained variance were automatically calculated by Roche LightCycler Software, showing a reliable qPCR efficiency of 1.945 (with slope of 0.346) and R^2^ of 0.992 (with error of 0.00716). According to the absolute standard curve of qPCR analysis, it was measured that RFP lentivirus contained 2.71 ± 0.31 × 10^8^ copy number/μL, whereas NIADS lentivirus contained 1.705 ± 0.165 × 10^8^ copy number/μL (Figure 1D). In the following experiment, we infected one copy number virus/MDA-MB-231 cell one-fold to evaluate the virus transfection efficiency between RFP and NIADS lentivirus. To do this, we transfected RFP lentivirus with different concentrations (from six-fold to 240-fold) on MDA-MB-231 cells. After three days post transfection, the MDA-MB-231 cells were measured for RFP positive percentage through flow cytometry (Figure 1F). With higher lentivirus transfection, RFP positive MDA-MB-231 cell numbers gradually increased in the virus infection and reached a maximum at 240-fold (red). We next compared the values of RFP positive MDA-MB-231 cell percentage and the lentivirus inputs (Figure 1G): the line curve showed 120-fold virus infection reached around 80% of all cell population, whereas 240-fold virus input increase to a near 100% transfection efficiency in MDA-MB-231 (termed as multiplicity of infection (MOI) = 1). The MDA-MB-231 cells were then transfected by different MOI of NIADS lentivirus for monitoring protein expression (Figure 1H). The immunoblotting assay clearly shows that, with an increasing MOI of NIADS virus, the fusion protein expression gradually increased and reached the maximum level at MOI = 3 or MOI = 6, using luciferase against antibody. This data implies that using MOI = 3 of pepAB virus would be effective enough for non-invasive caspase-3 reporter assay on MDA-MB-231 cells.

### 2.2. HDACi Induces Cell Death in TNBC and HER2-Enrich Breast Cancer Subtypes

**Figure 2:**
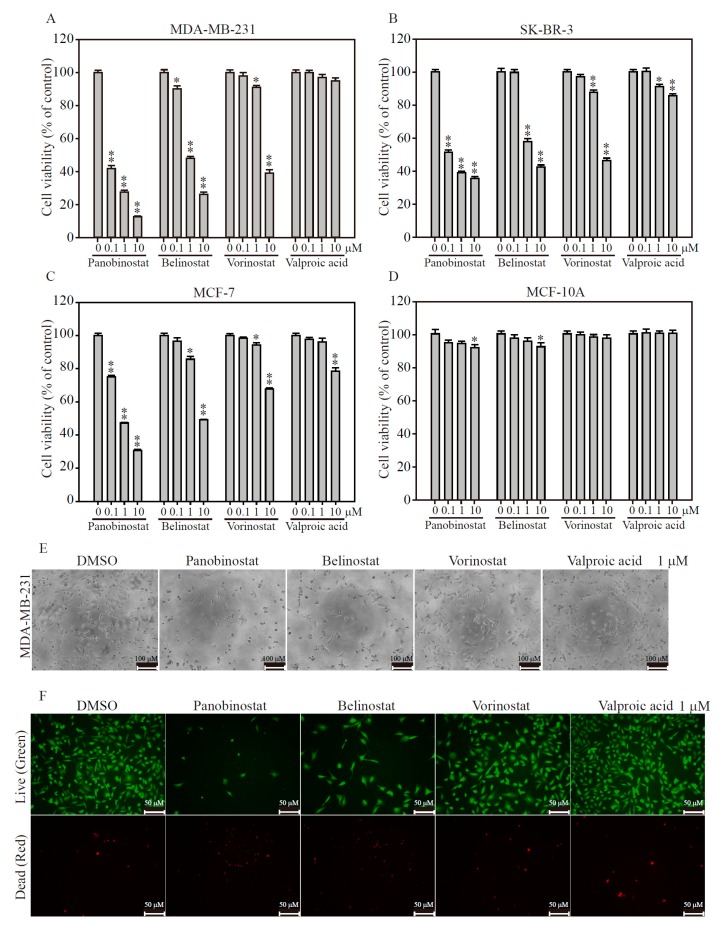
HDACi inhibits breast cancer cell viability and enhances cell apoptosis. Dose-dependent manner of 0.1, 1 and 10 μM Panobinostat, Belinostat, Vorinostat and Valproic acid inhibits (**A**) MDA-MB-231; (**B**) SK-BR-3; (**C**) MCF-7 breast cancer cells and (**D**) normal breast epithelia cells viabilities for 48 h by MTT assay determination; (**E**) The cell morphology change of 1 μM HDACi treatment for 48 h on MDA-MB-231 cells. Scale bars: 100 μM; (**F**) Fluorescence images of 1 μM HDACi exposed MDA-MB-231 cells for 48 h and determined by LIVE/DEAD viability/cytotoxicity assay. The cells were co-stained with 1 μM calcein-AM/10 μM PI and excited with light of 488 nm (green emission) to show viable cells. The same image of the cells was also excited with light of 532 nm, (red emission) to show the dead cells. Scale bar: 50 μM. Data are presented as the mean and standard error. Data were analyzed with Student’s *t*-test; all *p*-values were two-sided. *p*-values less than 0.05 are indicated with an asterisk, less than 0.01 is presented with two asterisks.

To understand whether or not epigenetic regulation via remodeling of chromatin may be used as an anti-cancer therapy in breast cancers, we evaluated four FDA-approved HDACi in luminal, HER2-enriched and TNBC breast cancer subtypes, as well as breast epithelial normal cell. By using Panobinostat, Belinostat, Vorinostat and Valproic acid dose-dependently to measure breast cancer cell viability for 48 h, we found Panobinostat and Belinostat significantly inhibited cell growth on MDA-MB-231 cells (TNBC, Figure 2A), rather than SK-BR-3 (HER2-enriched, Figure 2B) and MCF-7 (luminal, Figure 2C) cells. In addition, Panobinostat treatment illustrates dramatic drug sensitivity of IC50 cell growth on MDA-MB-231, SK-BR-3 and MCF-7 breast cancer cells with 0.024, 0.117 and 0.778 μM, respectively (Table 1). On the other hand, Belinostat has only shown significant values of cell growth inhibition on MDA-MB-231 and SK-BR-3 with 0.9 and 1.41 μM, respectively. It is worthwhile to note that all HDACi exposures demonstrated no cell growth effects on MCF-10A (breast epithelial normal cell, Figure 2D), making it an excellent and normal cell cytotoxicity-free candidate for breast cancer treatment in vitro and in vivo. We next examined the cell death event involved in HDACi treatment on MDA-MB-231 cells. As shown in Figure 2E, the cell numbers are significantly reduced in Panobinostat and Belinostat exposure for 24 h, as well as the cellular shrinkage is presented by cell morphology observation, compares to DMSO, Vorinostat and Valproic acid treatments. In addition, using live/dead assay, the results of the calcein AM (green) and PI (red) double staining under fluorescence microscopy showed that a majority of the cells survived under 1 μM of DMSO, Vorinostat and Valproic acid treatments for 24 h (Figure 2F). However, significant proportions of the cells (red) death are presented with 117 ± 10.4 and 107 ± 7 cells when MDA-MB-231 cells exposed to 1 μM Panobinostat and Belinostat, compares to 10 ± 1.7 cell death in DMSO treatment (Supplementary Figure S1). And furthermore, using flow cytometry to measure cell death occurred by HDACi exposure, we found Panobinostat, Belinostat and Vorinostat significantly induced sub-G1 (apoptotic cells) cell population with 50%, 43.2% and 40.5% of all cells, respectively (Supplementary Figure S2). These observations show that only Panobinostat and Belinostat strong activate anti-cancer effects on breast cancer cells, with superior cell growth inhibition and cell death induction in MDA-MB-231 and SK-BR-3 cells, indicating these drugs may be used as therapeutic drugs in TNBC and HER2-enrich breast cancer subtypes.

### 2.3. HDACi Induces Apoptosis and Cell Cycle Regulation through Histone Modification 

**Figure 3:**
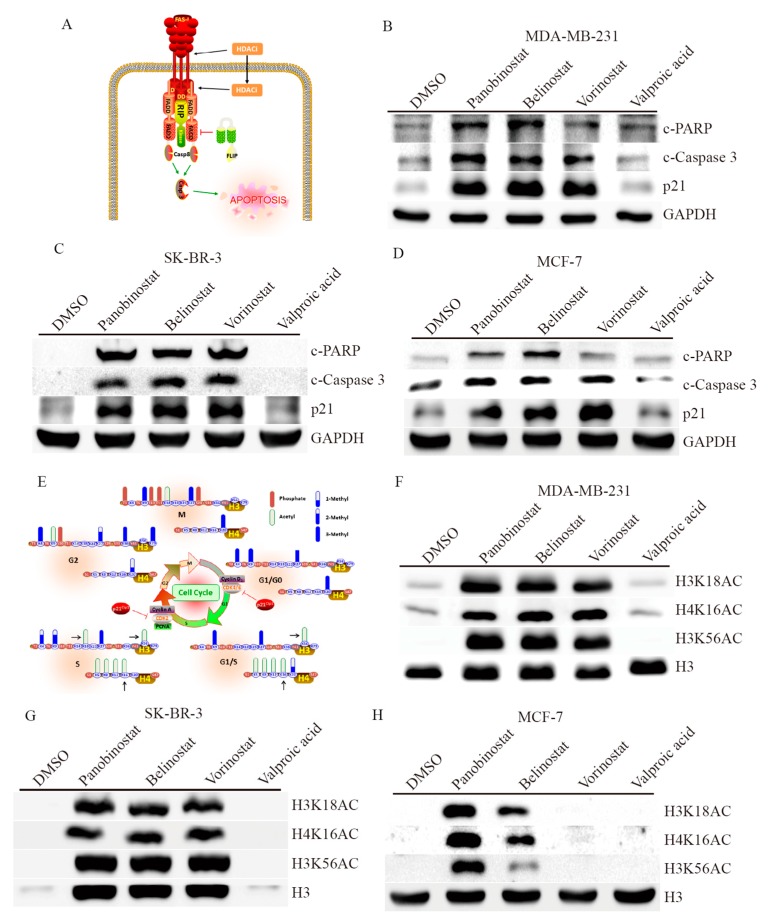
HDACi inhibits breast cancer cell viability and enhances cell apoptosis. (**A**) Schematic representation of the mechanism of HDACi induces apoptosis event through Fas ligand. The apoptotic biomarkers of PARP (Poly ADP-ribose polymerase), caspase-3 and P21 activation were triggered by 10 μM HDACi exposed on (**B**) MDA-MB-231, (**C**) SK-BR-3 and (**D**) MCF-7 breast cancer cells by western-blot analysis. (**E**) Schematic representation of the cell cycle impacts chromatin architecture on histone acetylation sites, whereas the regulation of P21 and downstream cell cycle proteins are indicated. The key histone acetylation sites of H3K18, H4K16 and H3K56 were determined by 10 μM HDACi exposures on (**F**) MDA-MB-231, (**G**) SK-BR-3 and (**H**) MCF-7 breast cancer cells by western-blot analysis. HDACi drugs were using Panobinostat, Belinostat, Vorinostat and Valproic acid. GAPDH (Glyceraldehyde 3-phosphate dehydrogenase) and H3 expressions are served as internal control.

Previous studies have shown that the use of HDACi forced cancer cell arrest either at the G1 or G2 phases of the cell cycle, resulting in cell growth inhibition and an increase in the percentage of apoptotic cells through both the death-receptor and intrinsic apoptotic pathways activation [13,14,15]. This apoptosis mechanism induced by HDACi enhances Fas-associated death domain protein (FADD) recruitment to TRAIL receptor (Tumor necrosis factor-related apoptosis-inducing ligand, DR4) in the death-inducing signaling complex, such as activation of membrane-proximal activator caspases (caspase-8) and effector caspases (caspase-3) (Figure 3A) [16]. To confirm that, we measured apoptotic markers in breast cancer cells with HDACi addition. In the presence of 1 μM of HDACi for 24 h, MDA-MB-231 showed a significant cleavage form of PARP (c-PARP) and caspase 3 (c-caspase-3) with Panobinostat, Belinostat and Vorinostat treatments (Figure 3B), compares to DMSO treatment. Furthermore, this apoptosis induction is also observed in SK-BR-3 (Figure 3C) and MCF-7 (Figure 3D) cells, but with less effectiveness in the Vorinostat treatment on MCF-7 cells. Beyond that, a cell cycle repressor protein, p21, was also significantly induced in the presents of Panobinostat, Belinostat and Vorinostat on breast cancer cells, which confirms the previous finding that HDACi forced cancer cell cycle arrest. Next, to investigate how HDACi impacts chromatin architecture and cell cycle regulation, we generated a schematic diagram of the effective H3 and H4 modifications during cell cycle progress (Figure 3E) [17,18]. In chromatin modification, phosphorylation, acetylation and different levels of methylation are playing important roles in cell cycle regulation. Since p21 presents outstanding enhancement in HDACi addition, showing p21 may inhibit the kinase activities of both cyclin dependent kinase 4/6 and cyclin dependent kinase 2, resulting in G1 and S phase cell cycle arrest, respectively. Thus, in this study, we selected three major histone acetylation sites that might mediate G1 and S phase cell cycle arrest in HDACi exposure, whereas the acetylation sites of H3K56 and H4K16 are presented in G1/S phase, and the acetylation site of H3K18 is additionally presented in S phase of cell cycle. The immunoblotting assay shows Panobinostat, Belinostat and Vorinostat strongly acetylate the sites of H3K18, H3K56 and H4K16 histone proteins on MDA-MB-231 (Figure 3F) and SK-BR-3 (Figure 3G) breast cancer cells. However, only Panobinostat acetylates H3K18, H3K56 and H4K16 histone proteins, whereas Belinostat weakly acetylates these three histone sites on MCF-7 (Figure 3H) cells. These data demonstrate that HDACi treatment induced breast cancer apoptosis or cell cycle arrest may be mediated from transcriptionally activation of CDKN1A gene by H3 and H4 modifications.

### 2.4. Validation of the papAB Bioluminescence Apoptosis Reporter In Vitro

**Figure 4:**
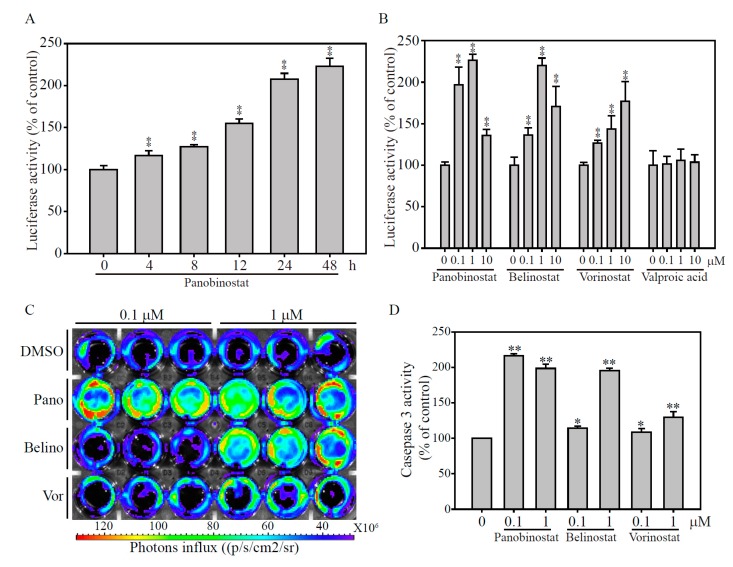
HDACi-induced apoptosis through NIADS assay. (**A**) A time-dependent manner (from 4 to 48 h) of Panobinostat treatment on NIADS-MDA-MB-231 cells. The cells received 1 μM of Panobinostat and their luciferase activity was measured. (**B**) Dose-dependent manner (from 0.1 to 10 μM) of HDACi treatments on NIADS-MDA-MB-231 cells. The cells received HDACi for 24 h tand heir luciferase activity was measured. NIADS-MDA-MB-231 cells treated with DMSO or HDACi from 0.1 and 1 μM for 24 h were detected with their luciferase activity (present by photos influx). (**C**) The IVIS image clearly shows that the luciferase activity is predominantly higher in Panobinostat and 1 μM of Belinostat treatments. (**D**) The photo influx calculation from the IVIS image was demonstrated. Data are presented as the mean and standard error. Data were analyzed with Student’s *t*-test; all *p*-values were two-sided. *p*-values less than 0.05 are indicated with an asterisk, less than 0.01 is presented with two asterisks.

To evaluate the utility of the NIADS in an in vitro platform, a stable NIADS fusion protein expression MDA-MB-231 (NIADS-MDA-MB-231) was cloned and expanded in normal culture condition. In a time-dependent bioluminescence activity observation, NIADS-MDA-MB-231 treated with 1 μM Panobinostat significant induced caspase-3 cleavage from 4 h and reached a maximum level at 2.3 folds post Panobinostat addition, compared to the group without Panobinostat addition (Figure 4A). Note that all bioluminescence activities from Panobinostat treatment were normalized to DMSO treatment group. Next, we wanted to know if drug concentration would be an issue affecting the result of NIADS assay. The HDACi dose-dependent treatments from 0.1 to 10 μM on NIADS-MDA-MB-231 for 24 h showed that the bioluminescence activity was induced in all Panobinostat, Belinostat and Vorinostat exposure groups (Figure 4B). However, it was also clearly showed that the bioluminescence activities from the groups of with 10 μM of Panobinostat and Belinostat treatment did not stand at the highest level, compared to 0.1 and 1 μM of Panobinostat and Belinostat treatments. The reason for this false-negative or dose-independent manner of apoptosis detection assay may be due to the high cytotoxicity of cancer drugs, causing too few cells to be determined for bioluminescence activity. Thus, it is suggested that, to monitor cell population after drug treatment, 20% to 30% remaining cells would be required to avoid the bias result.

To visualize the caspase-3 activation, we used the IVIS image system to measure luciferase activity after HDACi treatment on NIADS-MDA-MB-231 cells (Figure 4C). The bioluminescence data clearly showed that Panobinostat with 0.1 and 1 μM, Belinostat with 1 μM treatment for 24 h significantly induced Caspase 3 activation, compares to DMSO control treatment. After calculating the bioluminescence values, we found luciferase activities were significantly induced in Panobinostat, Belinostat and Vorinostat exposure groups, especially in 1 μM treatments of HDACi (*p* < 0.001), whereas only Panobinostat with low concentration (0.1 μM) still remain high cytotoxicity on NIADS-MDA-MB-231 cells, compares to DMSO control (Figure 4D). 

### 2.5. HDACi and Doxorubicin Combination Synergically-Induced Apoptosis Event

**Figure 5:**
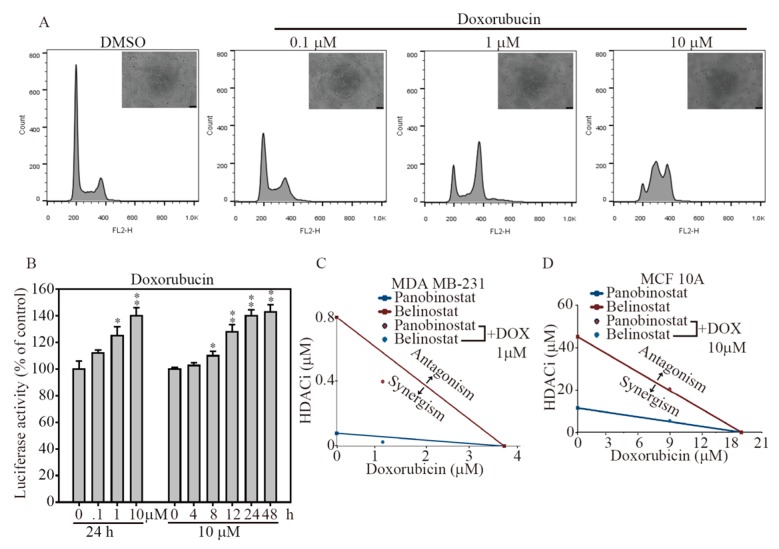
HDACi synergiclly enhances anti-cancer activity of doxorubicin through NIADS assay (**A**) The cell cycle analysis of doxorubicin treatments from 0.1 to 10 μM on MDA-MB-231 cells by flow cytometry determination. The cell morphology changes by doxorubicin exposure were also demonstrated; (**B**) A dose-dependent manner (from 0.1 to 10 μM) and a time-dependent manner (from 4 to 48 h) of doxorubicin treatment on NIADS-MDA-MB-231 cells were evaluated for their luciferase activity. Combination Index (CI) values of Panobinostat/Belinostat and doxorubicin treatment on (**C**) MDA-MB-231 and (**D**) MCF10A IC50. The red dot and blue dot represent the synergistic effects of doxorubicin treatment with 1 µM Panobinostat and Belinostat, respectively. Data are presented as the mean and standard error. Data were analyzed with Student’s *t*-test; all *p*-values were two-sided. *p-*values less than 0.05 are indicated with an asterisk, less than 0.01 is presented with two asterisks.

To reveal whether HDACi confers anti-cancer activity with chemotherapy in breast cancer therapy, we measured the cell cycle distribution and cell morphology changes in a dose-dependent treatment of doxorubicin for 24 h on MDA-MB-231 cells (Figure 5A). The data illustrated that 10 μM doxorubicin treatment significant arrested the cell cycle at S-phase, whereas DMSO, 0.1 and 1 μM doxorubicin treatments had no significant change on MDA-MB-231 cells. In a time- and dose-dependent bioluminescence activity observation, NIADS-MDA-MB-231 treated with 1 and 10 μM doxorubicin significant induced caspase-3 cleavage from 8 h and reached a maximum level at 24 or 48 h post doxorubicin addition, compared to DMSO addition group (Figure 5B). To examine whether the HDACi and doxorubicin combination was additive or synergistic, we used the approach designed by Chou and Talalay to calculate the combination index (CI) [19]. As shown in Figure 5C, the IC50 cell survival concentrations of MDA-MB-231 breast cancer cells were measured after both mono (Panobinostat at 0.08 μM, Belinostat at 0.8 μM and doxorubicin at 3.65 μM) and combination (Panobinostat at 0.025 μM and Belinostat at 0.4 μM with 1.2 μM doxorubicin) drug exposure; the calculated CIs (Panobinostat at 0.586, Belinostat at 0.774) were less than one for the selected concentrations, demonstrating that the combination of both HDACi and doxorubicin elicited a synergistic effect on cellular proliferation inhibition. In addition, the synergism of HDACi and doxorubicin combination was also performed on normal breast epithelia MCF-10A cells. Under the same treatment condition, the IC50 cell survival concentrations were measured after both mono (Panobinostat at 13 μM, Belinostat at 43.2 μM and doxorubicin at 18.6 μM) and combination (Panobinostat at 9.6 μM and Belinostat at 0.4 μM with 9 μM doxorubicin) drug exposure; the calculated CIs (Panobinostat at 1.033, Belinostat at 1.016) were equal to one for the selected concentrations, demonstrating that the both HDACi and doxorubicin combination elicited a additive effect on cellular proliferation inhibition on normal breast cells. This highly cytotoxicity effect on breast cancer cells makes the mono or combination therapy based on HDACi an excellent anti-cancer drug during breast cancer treatment.

## 3. Discussion

Apoptosis is important step in embryonic development, tissue homeostasis, and removal of cells with DNA lesions or other types of injuries [20,21], whereas protein analysis or genomics screening are the most-used techniques to unravel apoptotic signaling cascades in the context of toxicant-induced cell death at an end-point observation [20,22]. To generate meaningful data with the required throughput, high-quality assay methods are needed to translate specific biomolecular phenomena into observable parameters. The recent advent of high-content live-cell imaging technologies has provided researchers with the ability to visualize cellular phenotypes in high-throughput multi-well formats [23,24]. Frequently, these assays are accomplished using fluorescent reporters and analyzed to provide kinetic data for the duration of the experiment. However, assays based on fluorescent protein activation are commonly used, but they impose limits on assay design and miniaturization since they typically provide low sensitivity. Photon emission application such as bioluminescence is by far the dominant assay methodology due to its broad adaptability to biological targets and automation methods, and its ability to deliver the speed, accuracy, and sensitivity necessary for successful high through put campaigns [25,26]. Collectively, our study used new capabilities of bioluminescence technologies as a reporter for live-cell apoptosis detection and will provide perspectives on future developments in anti-cancer drug development both in vitro and in vivo. Thus, an appropriate and optimized NIADS-based animal platform should be built before accessing pre-clinical trial from anti-cancer drug in vivo validation.

Activation of the caspase-3 pathway is a hallmark of apoptosis and can be used in cellular assays to quantify activators and inhibitors of the “death cascade.” The active form of caspase-3 has been implicated as an “effector” caspase associated with the initiation of the “death cascade” and is therefore an important marker of the cell’s entry point into the apoptotic signaling pathway [27]. Each methodology has its unique advantages and disadvantages [28]. Cell morphology and DNA-based experiments are inexpensive methods for detection of apoptotic cells and they are fairly reliable. On the other hand, quantitative apoptosis measurement lacks objectivity and reproducibility. Annexin V affinity assay and TUNEL (Terminal deoxynucleotidyl transferase dUTP nick end labeling) dye labeling for apoptotic cells are the most commonly used methods for apoptosis event quantification, but the techniques are time-consuming as they have complicated steps and sometimes require live cells to perform assays. Caspase activity with either colorimetric, fluorometric or luminescent determinations is the most expensive option among all apoptosis detection methodologies, but it provides easy, rapid and quantitative apoptosis measurement in multiple-well plates. In this study, NIADS provides not only the apoptosis event quantification in multiple-well plate, but also includes a single step of virus transduction before drug exposure, which makes NIADS a rapid, easy and inexpensive real-time apoptosis measurement tool. Like many other apoptosis assays, the limitation of NIADS is that it can only be used for caspase-3-dependent apoptosis detection. The previous study indicated that multiple direct and indirect interactions are overlapped and interacted between the apoptosis and autophagy regulations during cell death [29]. The majority of these interactions have apoptosis altering autophagy, whereas the activated caspase-8/9 connects autophagy-related proteins (ATGs) or Beclin-1 molecules and turns on autophagy process [30]. To extend the application of NIADS for other types of cell death, we have generated the LC3-dependent detection for further autophagy measurement through lentivirus transduction.

The quantification of apoptotic cell death is an integral component of exploring cell biology, responses to cellular stress and performing high-throughput drug screens [31]. Annexin V/FITC assay is the most chosen technique, detecting and quantifying cell apoptosis events, usually accomplished by flow cytometry assessment. However, this cell-based measurement requires extensive sample handling and a great deal of labor for cell harvesting and analysis. More importantly, Annexin V/FITC assay cannot be used as a high-throughput apoptosis detection platform, due to the complexity of its probe labeling and apoptotic cell detection. In our developed NIADS assay, which involves the advantages of relatively low cell numbers, trace amounts of bioluminescence, short assay times and ease-of-handling, apoptosis detection assay is made suitable for high-throughput cell-based screening of drug libraries and related applications. The time frame and performance of the cell viability assays described here are optimal for many screening applications, allowing quantitative parallel assessment of hundreds of samples per hour. For example, in a typical primary screening of mono or combinatorial cancer drugs, test cells are treated with different chemical compounds and changes in their viability monitored periodically at 24 h. With the appropriate optimization, this cell-based detection might be amenable to automation and allow substantial savings in labor and assay costs through miniaturization of cell input (multiple-well plates). 

According to previous studies, either altered acetylation levels or HDAC enzyme dysfunctions are directly linked to human malignant cancers. Furthermore, HDAC was found to predominantly express in hematological cancers and solid tumors, and is correlated with a poor prognosis. So far, 18 human HDAC, divided into two families, have been identified based on their homology to yeast HDAC, whereas HDAC class I and class II are responsible for inducing cancer cell proliferation, and tumor angiogenesis suppressing cell apoptosis events [32]. Vorinostat is the most advanced of the HDACi in clinical development. Vorinostat was the first HDACi approved by Fthe DA for clinical use in treating patients with advanced cutaneous T-cell lymphoma [33]. Later, a hydroxamic acid-based HDACi including Belinostat has also been approved in 2014 by the FDA to treat peripheral T-cell lymphoma. Moreover, Panobinostat has somehow proved more potent than Vorinostat for multiple myeloma and solid tumors and was approved on 28 August 2015 by the European Medicines Agency. In this study, we measured the cell viability and cancer apoptosis event of currently-used HDACi in clinic on breast cancers, with the result that both Panobinostat and Belinostat have great potential for application in TNBC and HER2-enriched subtype breast cancer therapy, without cytotoxicity on normal breast epithelia cells. 

## 4. Materials and Methods

### 4.1. Cell Lines and Culture Conditions

Human mammary gland epithelial adenocarcinoma cell line MDA-MB-231 and human kidney epithelial Phoenix-ECO cells were purchased from the American Tissue Culture Collection (ATCC, Manassas, VA, USA) and maintained in Dulbecco’s Modified Eagle Medium: Nutrient Mixture F-12 (DMEM/F-12) Media (Gibco, CA, USA). The cells were incubated with 10% (*v*/*v*) fetal bovine serum (FBS, Biological Industries, Kibbutz Beit Kaemek, Israel). The supplement of 100 units/mL penicillin and 100 mg/mL streptomycin were used and cultured in a 37 °C incubator with 5.0% CO_2_. The medium was replaced every two days, and when cells reached 80% confluence, they were passaged using 0.25% trypsin/EDTA (Gibco, CA, USA).

### 4.2. Lentiviral Production of Non-Invasive Apoptosis Detection Sensor (NIADS) and Cell Transduction

The fragments of papA (5′-ATGAACGAAGCATATGTACATGACGGTCCTGTACGCTCACTGAAC-3′) and pepB (5′-AAGGCACGAAAGGAAGCAGAACTGGCAGCAGCAACTGCAGAACAG-3′) were constructed in split luciferase PLAS3.1-neo lentivirus plasmid with 3X caspase 3 cleavage sequences (5′-GACGAAGTCGATGACGAAGTCGATGACGAAGTCGAT-3′). The NIADS construct or the RFP (red fluorescent protein) plasmid was co-transfected with pMD2.G (Addgene plasmid #12259) and psPAX2 (Addgene plasmid #12260, both kindly provided by Didier Trono, EPFL, Lausanne, Switzerland). Lentiviral particles were collected at 36 and 72 h and then concentrated with a Lenti-X Concentrator^®^ (Clontech, Mountain View, CA, USA). The lentivirus concentration for each gene was quantified by qPCR. Biohazards and restricted materials were used in this study in accordance with the “Safety Guidelines for Biosafety Level 1 to Level 3 Laboratory”. The protocol was approved by the Institutional Biosafety Committee at Taipei Medical University, Taipei, Taiwan. The lentivirus concentration for each gene was quantified by qPCR. MDA-MB-231 cells were infected in a 6-cm dish, with each well containing 1 × 10^6^ cells and indicated concentrations of lentivirus for 48 h. 

### 4.3. Transfection and Cell Line Selection 

MDA-MB-231 cells were transfected with pcDNA3 plasmids expressing the firefly luciferase gene (the gene sequences were originally from *luc* 4.1; Chris Contag, Stanford University, Stanford, CA, USA), as described previously [34]. Briefly, 5 × 10^6^ MDA-MB-231 cells were washed twice with phosphate buffered saline (PBS) and mixed with 10 μg of plasmid. Two pulses were applied for 20 milliseconds under 1.2 kV on the pipette-type MicroPorator MP-100 (Digital Bio, Seoul, Korea). The stable cells were selected 48 h later with G418 (6 mg/mL).

### 4.4. Real-Time Quantitative Polymerase Chain Reaction (qPCR) 

Primers for the *WPRE* (Woodchuck Hepatitis Virus Response Element) region (forward 5′-TCATGCTATTGCTTCCCGTA-3′ and reverse 5′-CCAAGGAAAGGACGATGAT-3′) were used for lentivirus quantification. All oligo primers were synthesized by Genomics BioSci and Tech (Taipei, Taiwan). A LightCycler thermocycler (Roche Molecular Biochemicals, Mannheim, Germany) was used for qPCR analysis. One microliter of sample and master mix (Roche FastStart Universal SYBR Green Master Mix, Roche Applied Science, Mannheim, Germany) was first denatured for 10 min at 95 °C and then subjected to 40 cycles (denaturation at 95 °C for 5 s; annealing at 60 °C for 5 s; and elongation at 72 °C for 10 s) with detection of fluorescence intensity. All the PCR samples underwent a melting curve analysis to detect non-specific PCR products. Luciferase gene expression from the qPCR analysis was normalized to *GUS* (β-glucuronidase) expression as an indicator of DNA input using the built-in Roche LightCycler Software, version 4 (Roche Molecular Biochemicals, Mannheim, German).

### 4.5. Absolute qPCR

To generate an absolute quantitative standard curve for qPCR analysis, we cloned the PCR product of the human GUS gene into the TA cloning kit (RBC Bioscience; Taipei, Taiwan), which was purchased from Genomics BioSci and Tech (Taipei, Taiwan). After gene sequencing, E. coli amplification, plasmid purification, and molecular weight determination, the copies of the GUS gene were calculated and diluted from 10^8^ to 10^2^ per μL. Each copied gene was measured for accuracy and a linear correlation. The qPCR efficiency analysis for standard curve is calculated by the formula of Efficiency = 10^−1/slope^, whereas qPCR unexplained variance of R^2^ value was calculated by the formula of R^2^ = 1 − Error.

### 4.6. Protein Extraction, Western Blotting, and Antibodies

For western blot analysis, MDA-MB-231 cells were washed once with ice-cold PBS and lysed with radioimmunoprecipitation assay (RIPA) lysis buffer containing protease inhibitors. Fifty micrograms of protein from each sample was resolved by sodium dodecyl sulfate polyacrylamide gel electrophoresis (SDS-PAGE) and transferred to a nitrocellulose membrane. The anti-GAPDH (sc-32233) was purchased from Santa Cruz Biotechnology (Santa Cruz, CA, USA); the anti-P21 (GTX629543) antibody was purchased from GeneTex Inc. (Irvine, CA, USA); and the anti-H3 (ab1791), H3K18AC (ab1191) and H3K56AC (ab76307) were purchased from Abcam plc (Cambridge, UK); and anti-H4K16AC (CS204361) antibody was purchased from Millipore (Billerica, MA, USA); and anti-PARP (Poly (ADP-ribose) polymerase, #9532) antibodies were purchased from Cell Signaling Technology (Danvers, MA, USA). The secondary anti-mouse and anti-rabbit antibodies were purchased from Santa Cruz Biotechnology. All primary antibodies were used at a 1:1000 dilution with overnight hybridization, followed by one-hour incubation with a 1:4000 dilution of the secondary antibodies.

### 4.7. MTT Viability Assay

MDA-MB-231 cells were seeded in 96-well plates and incubated with HDACi or doxorubicin for 48 h. MTT (3-(4,5-Dimethylthiazol-2-yl)-2,5-diphenyltetrazolium bromide) reagent was added in medium for two hours and assayed according to the manufacturer’s protocol. Absorbance was monitored at 570 nm and background was monitored at 630 nm using an ELISA plate reader. Final absorbance units were computed by background subtraction.

### 4.8. Live/Dead Cell Assay

MDA-MB-231 cells were seeded in 12-well plates overnight and incubated with HDACi for 48 h in the normal culture condition. The medium of the cells was removed and treated for 30 min in the dark with 1 μM calcein-AM and 10 μM Propidium iodide (PI) prepared in normal culture medium. The fluorescence images of Live were captured under light wavelength of 488 nm (green emission) to show viable cells. The same image of the cells was also excited with light wavelength of 532 nm, (red emission) to show the dead cells.

### 4.9. Cellular Bioluminescence Assay

Live cells were plated and treated with HDACi or doxorubucin for indicated time and drug concentration. d-luciferin (1.5 mg/mL in PBS) with 1X MTT reagent was added to each well and photon counts were collected by a Xenogen IVIS CCD camera (Xenogen, Alameda, CA, USA) system soon after luciferin addition. The cells were placed in normal culture condition for 2 h and performed MTT assay. The photon count from each well was than normalized with its MTT data for cellular bioluminescence assay.

### 4.10. Bioluminescence (IVIS) Imaging

Bioluminescence imaging was performed with a highly sensitive, cooled CCD camera mounted in a light-tight specimen box (In Vivo Imaging System—IVIS; Xenogen). The multiple well plate was exposure with d-luciferin (1.5 mg/mL) and placed on a warmed stage inside the camera box during imaging. The light emitted from the cells was detected by the IVIS camera system, integrated, digitized, and displayed. Regions of interest on the displayed images were identified, and the total photon count were quantified using Living Image^®^ software 4.0 (Caliper, Alameda, CA, USA). 

### 4.11. Flow Cytometry Analysis 

MDA-MB-231 (1 × 10^6^ cells/dish) were plated in 6-cm dishes for infection. An equal number of virus particles and MDA-MB-231 cells was defined as 1-fold. MDA-MB-231 cells were exposed to different folds of concentrated virus harboring the RFP plasmid. Lentivirus-transduced cells were harvested three days after infection, and the RFP-positive cell population was analyzed by flow cytometry (FACSCalibur, BD Biosciences, San Jose, CA, USA).

### 4.12. Statistical methods

All data were expressed as mean ± error, and student *t*-test analysis was performed for the pairwise samples. All statistical comparisons were performed using SigmaPlot graphing software (San Jose, CA, USA) and Statistical Package for the Social Sciences v.13 (SPSS, Chicago, IL, USA). A *p*-value < 0.05 was considered statistically significant, and all statistical tests were two-sided.

## 5. Conclusions

NIADS assays have proven to work reliably with mammalian cells in assessing their viability, cell numbers, and drug/effector action. This assay is compatible with standard microplates and commercial prompt and time-resolved luminescent plate readers. In this manuscript, we showed both traditional apoptotic marker investigation and NIADS assay clearly identify that a low dose of Panobinostat and Belinostat effectively activate apoptosis events in breast cancer cells, whereas NIADS is much easier and more reliable at providing a positive and kinetic signal response with the addition of substrate and photon counting. In addition, a synergic anti-breast cancer effect was also found between HDACi and doxorubicin on MDA-MB-231 cells, indicating a combination therapy of these drugs might be applied in clinic for advanced-stage breast cancer patients.

## Figures and Tables

**Table 1:** The IC50 of cell viability by HADCi treatments on breast cancer and normal epithelia cells.

Cell Line	Panobinostat	Belinostat	Vorinostat	Valproic Acid
MDA-MB-231	0.024	0.9	7.87	>10
SK-BR-3	0.117	1.41	8.94	>10
MCF-7	0.778	9.64	>10	>10
MCF-10A	>10	>10	>10	>10

Drug concentration is presented in μm.

## References

[1] Li Y., Seto E. (2016). HDACs and HDAC Inhibitors in Cancer Development and Therapy. Cold Spring Harb. Perspect. Med..

[2] Yen C.Y., Huang H.W., Shu C.W., Hou M.F., Yuan S.S., Wang H.R., Chang Y.T., Farooqi A.A., Tang J.Y., Chang H.W. (2016). DNA methylation, histone acetylation and methylation of epigenetic modifications as a therapeutic approach for cancers. Cancer Lett..

[3] Rekowski M., Giannis A. (2010). Histone acetylation modulation by small molecules: A chemical approach. Biochim. Biophys. Acta.

[4] Liang G., Weisenberger D.J. (2017). DNA methylation aberrancies as a guide for surveillance and treatment of human cancers. Epigenetics.

[5] Waldmann T., Schneider R. (2013). Targeting histone modifications—Epigenetics in cancer. Curr. Opin. Cell Biol..

[6] Losson H., Schnekenburger M., Dicato M., Diederich M. (2016). Natural Compound Histone Deacetylase Inhibitors (HDACi): Synergy with Inflammatory Signaling Pathway Modulators and Clinical Applications in Cancer. Molecules.

[7] Torre L.A., Bray F., Siegel R.L., Ferlay J., Lortet-Tieulent J., Jemal A. (2015). Global cancer statistics, 2012. CA Cancer J. Clin..

[8] Perou C.M., Sorlie T., Eisen M.B., van de Rijn M., Jeffrey S.S., Rees C.A., Pollack J.R., Ross D.T., Johnsen H., Akslen L.A. (2000). Molecular portraits of human breast tumours. Nature.

[9] Bardia A., Mayer I.A., Diamond J.R., Moroose R.L., Isakoff S.J., Starodub A.N., Shah N.C., O’Shaughnessy J., Kalinsky K., Guarino M. (2017). Efficacy and Safety of Anti-Trop-2 Antibody Drug Conjugate Sacituzumab Govitecan (IMMU-132) in Heavily Pretreated Patients With Metastatic Triple-Negative Breast Cancer. J. Clin. Oncol..

[10] Garcia-Teijido P., Cabal M.L., Fernandez I.P., Perez Y.F. (2016). Tumor-Infiltrating Lymphocytes in Triple Negative Breast Cancer: The Future of Immune Targeting. Clin. Med. Insights Oncol..

[11] Chatterjee S., Lesniak W.G., Gabrielson M., Lisok A., Wharram B., Sysa-Shah P., Azad B.B., Pomper M.G., Nimmagadda S. (2016). A humanized antibody for imaging immune checkpoint ligand PD-L1 expression in tumors. Oncotarget.

[12] Sellers W.R., Fisher D.E. (1999). Apoptosis and cancer drug targeting. J. Clin. Investig..

[13] Lakshmaiah K.C., Jacob L.A., Aparna S., Lokanatha D., Saldanha S.C. (2014). Epigenetic therapy of cancer with histone deacetylase inhibitors. J. Cancer Res. Ther..

[14] Zhang J., Zhong Q. (2014). Histone deacetylase inhibitors and cell death. Cell. Mol. Life Sci..

[15] Venugopal B., Evans T.R. (2011). Developing histone deacetylase inhibitors as anti-cancer therapeutics. Curr. Med. Chem..

[16] Inoue S., Harper N., Walewska R., Dyer M.J., Cohen G.M. (2009). Enhanced Fas-associated death domain recruitment by histone deacetylase inhibitors is critical for the sensitization of chronic lymphocytic leukemia cells to TRAIL-induced apoptosis. Mol. Cancer Ther..

[17] Ma Y., Kanakousaki K., Buttitta L. (2015). How the cell cycle impacts chromatin architecture and influences cell fate. Front. Genet..

[18] Rossetto D., Avvakumov N., Cote J. (2012). Histone phosphorylation: A chromatin modification involved in diverse nuclear events. Epigenetics.

[19] Chou T.-C., Talalay P. (1984). Quantitative analysis of dose-effect relationships: The combined effects of multiple drugs or enzyme inhibitors. Adv. Enzyme Regul..

[20] Elmore S. (2007). Apoptosis: A review of programmed cell death. Toxicol. Pathol..

[21] Pampfer S. (2000). Apoptosis in rodent peri-implantation embryos: Differential susceptibility of inner cell mass and trophectoderm cell lineages—A review. Placenta.

[22] Fan F., Wood K.V. (2007). Bioluminescent assays for high-throughput screening. Assay Drug Dev. Technol..

[23] Chalasani A., Ji K., Sameni M., Mazumder S.H., Xu Y., Moin K., Sloane B.F. (2017). Live-Cell Imaging of Protease Activity: Assays to Screen Therapeutic Approaches. Methods Mol. Biol..

[24] Specht E.A., Braselmann E., Palmer A.E. (2017). A Critical and Comparative Review of Fluorescent Tools for Live-Cell Imaging. Annu. Rev. Physiol..

[25] McCann T. (2010). Live cell imaging: An industrial perspective. Methods Mol. Biol..

[26] Joseph J., Seervi M., Sobhan P.K., Retnabai S.T. (2011). High throughput ratio imaging to profile caspase activity: Potential application in multiparameter high content apoptosis analysis and drug screening. PLoS ONE.

[27] Nicholson D.W., Ali A., Thornberry N.A., Vaillancourt J.P., Ding C.K., Gallant M., Gareau Y., Griffin P.R., Labelle M., Lazebnik Y.A. (1995). Identification and inhibition of the ICE/CED-3 protease necessary for mammalian apoptosis. Nature.

[28] Archana M., Bastian, Yogesh T.L., Kumaraswamy K.L. (2013). Various methods available for detection of apoptotic cells—A review. Indian J. Cancer.

[29] Thorburn A. (2008). Apoptosis and autophagy: Regulatory connections between two supposedly different processes. Apoptosis.

[30] Tsapras P., Nezis I.P. (2017). Caspase involvement in autophagy. Cell Death Differ..

[31] Gelles J.D., Chipuk J.E. (2016). Robust high-throughput kinetic analysis of apoptosis with real-time high-content live-cell imaging. Cell Death Dis..

[32] Yoon S., Eom G.H. (2016). HDAC and HDAC Inhibitor: From Cancer to Cardiovascular Diseases. Chonnam Med. J..

[33] Duvic M., Vu J. (2007). Vorinostat: A new oral histone deacetylase inhibitor approved for cutaneous T-cell lymphoma. Expert Opin. Investig. Drugs.

[34] Tu S.H., Hsieh Y.C., Huang L.C., Lin C.Y., Hsu K.W., Hsieh W.S., Chi W.M., Lee C.H. (2017). A rapid and quantitative method to detect human circulating tumor cells in a preclinical animal model. BMC Cancer.

